# Clinical experience of novel interconnected porous hydroxyapatite ceramics for the revision of tumor prosthesis: a case report

**DOI:** 10.1186/1477-7819-7-76

**Published:** 2009-10-21

**Authors:** Yukihiro Yoshida, Shunzo Osaka, Yasuaki Tokuhashi

**Affiliations:** 1 Department of Orthopedic Surgery, Nihon University School of Medicine, Tokyo, Japan; 2 Nerima Hikarigaoka Hospital, Nihon Univeristy 2-11-1, Hikarigaoka Nerima-ku, Tokyo, Japan

## Abstract

**Background:**

As for being cautious with tumor prostheses, revision of uncemented tumor prostheses in particular, it is necessary to remove cortical bone from the stem circumference with a chisel when the stem is extracted. This assures that bone in-growth will occur within the stem in itself. As a result, re-substitution of mass autogenous bone graft round a new stem is subsequently necessary. When rivision of uncemented tumor prosthesis of distal femur was performed, we evade fibula transplant by transplanting interconnected porous hydroxyapatite ceramic (IP-CA: Neobone) with a self bone, and reports its experience with the case that acquired enough strength.

**Case report:**

In this report, we present the case of a 27-year-old female with stem breakage of tumor prosthesis and do revision surgery for prosthetic failure. In the case of revision surgery, autologous bone and Neobone were mixed, and this was transplanted to stem circumference. The Radiological Evaluation System of the ISOLS showed excellent results for all items. She can walk without using a cane or orthosis, and the score of the MSTS is 80%.

**Conclusion:**

When revision of uncemented tumor prostheses of the distal femur was performed, we avoided fibula graft by using Neobone with the patient's own bone tissue. Our experience with this case may indicate that adequate strength is achieved.

## Background

In surgery for malignant bone tumors, the implantation of joint prostheses for tumors (tumor prostheses) after wide resection is an important reconstruction method. However, complications such as infection and prosthesis fracture develop in some cases [1,2]. To enhance bone strength around the stem of a femoral component, we used an interconnected porous hydroxyapatite ceramic (IP-CA: Neobone) in combination with an autogenous bone graft.

## Case report

**Figure 1:**
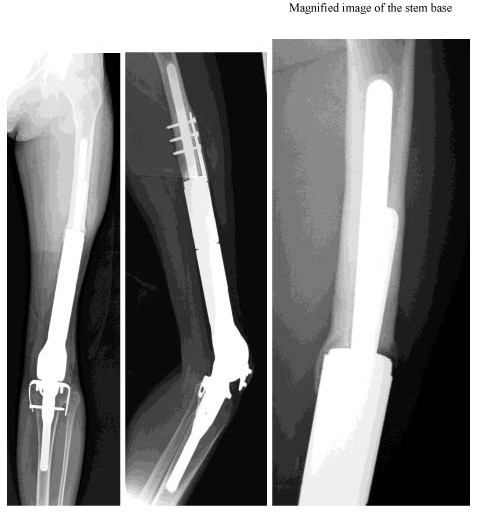
**Plain X-ray films at the time of stem fracture**.

**Figure 2:**
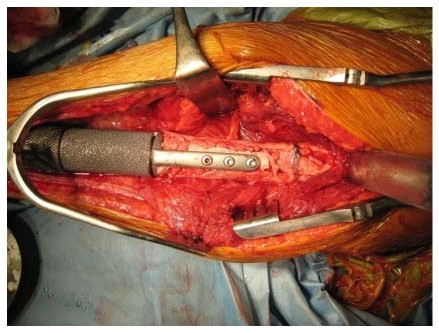
**Insertion of a new stem and adequate reinforcement with artificial and autogenous bone**.

**Figure 3:**
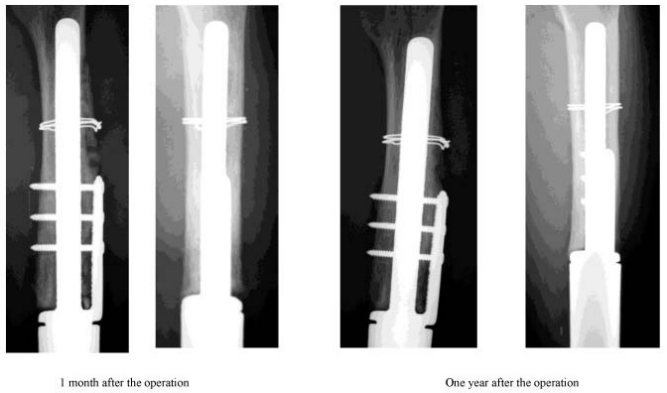
**Postoperative bone formation around the stem**. Granular forms were still observed 1 month after the operation, but the borders between IP-CA granules and between IP-CA and bone became unclear, and sclerotic changes were observed around the stem, suggesting adequate bone strength.

A 27-year-old female was referred to our hospital on May 16, 1996 and admitted due to a suspected malignant bone tumor in the left distal femur. On May 20, biopsy was performed. Histopathological examination demonstrated osteosarcoma, and preoperative chemotherapy was immediately performed according the chemotherapy protocol of our department for osteosarcoma. On September 25, wide resection was performed, and the affected limb was reconstructed using a tumor prosthesis (Howmedica Modular Reconstruction System: HMRS). Subsequently, postoperative chemotherapy was performed for about 6 months. On May 2007, about 11 years after the operation, she noticed pain in the left thigh during walking. Due to gradual aggravation of the pain, she visited our department on September 6, 2007. X-ray examination revealed fracture at the base of the stem of the femoral component (Fig. 1). We planned revision using a tumor prosthesis, and obtained a custom-made femoral component stem (diameter, 12 mm; length, 15 cm). Until the completion of the stem, a knee-ankle-foot orthosis was employed, and crutches were used for walking. The preoperative score of the Musculoskeletal Tumor Society was 60%. On November 7, 2007, revision surgery was performed. The wound was exposed using the previous skin incision. Bone formation at the stem base was good. Rectangular fenestration along the stem in the bone was performed in the proximal femur using a chisel, and the stem in the bone was removed. A new stem was inserted, and adequate grafting with Neobone and autogenous bone was performed around the stem. Only the bearing bush and femoral component were replaced with new ones, and the operation was completed (Fig. 2). Two weeks after the operation, passive range-of-motion training was initiated. Until 6 weeks after surgery, no weight bearing was performed. Seven weeks after the operation, walking training was initiated using a knee orthosis with gradual weight bearing. The knee orthosis was used until 6 months after the operation. At present, about one year after surgery, good bone formation around the stem is observed on plain X-ray films. Evaluation using The Implant Evaluation System of the International Symposium on Limb Salvage showed excellent results for all items (Bone remodeling, Interface, Anchorage) after as well as before the operation (Fig. 3). She can walk without using a cane or orthosis, and the score of the Musculoskeletal Tumor Society is 80%.

## Discussion

Due to recent advances in surgery and chemotherapy for primary malignant bone tumors, the survival rate has increased, and the usefulness of tumor prostheses as a limb reconstruction method has also been confirmed. However, complications such as infection associated with these prostheses and their loosening and fracture have presented problems. Concerning stem fracture, in 1994, R Capanna et al. reported stem fracture of modular uncemented tumor prostheses in 6 (6.3%) of 95 patients[2]. In 2001, Mittenmayer et al. reported major complications in 19 of 100 patients using uncemented tumor prostheses, consisting of 11 patients showing aseptic loosening and 4 each showing septic loosening and implant fracture [3]. In addition, in 2006, G Gosheger et al. reported stem fracture in 4 (1.6%) of 250 patients using uncemented tumor prostheses[4]. At our department, stem fracture has been observed in 5 patients, and the mean duration until the fracture was 4 years and 6 months (10 months-9 years). The system used was the KMFTR implant (Kotz Modular Femur and Tibia Reconstruction System) in 4 patients and the PH type 1 (Physio hinge type 1) in the remaining one. The stem diameter was 10 mm in 4 patients and 11 mm in 1. Both stems were relatively thin for the distal femur. After revision, the stem diameter was 10 mm in only 1 patient, and stems with a greater diameter than those in the previous operation were used in the others. The possible causes of stem fracture include improvement in patients' activity and stem loosening. In general, stem fracture is considered to be associated with the design (hinge-type structure) of prostheses themselves[5]. Some authors have recommended the use of relatively thick rather than thin stems[3,6]. Various tumor prostheses have been studied and developed by researchers, but optimal prostheses have not yet been produced[7].

Unlike conventional hydroxyapatite (HA) ceramics, IP-CA was developed employing a new concept with importance placed on interconnectivity among air pores. IP-CA also allows new bone to enter air pores in the deep area, and has adequate strength for clinical use[8]. Myoui et al. used IP-CA in 62 surgically treated patients with benign bone tumor, bone fracture, and inflammatory diseases, and observed favorable clinical results[9]. On X-ray films, the border between IP-CA granules and between IP-CA and bone became unclear due to marked osteosclerosis in 61% of the patients after 6 months. Concerning cautionary items at the time of filling during the operation, Nakase et al. described that the initial strength acquired is lower using IP-CA than cortical bone, and recommended that the initial strength should be made as high as possible by combining IP-CA with autogenous cortical bone in fragile areas such as the bone fracture area[10]. In our patients, for the reinforcement of the femoral stem, IC-PA was mixed with autogenous cortical bone to achieve maximal initial strength. On X-ray films, the borders between IP-CA and autogenous bone became unclear. X-ray rating by Myoui et al. was Grade 3.

## Conclusion

At the time of the revision of the tumor prosthesis, to enhance the strength of bone around the stem, we planned to use a large amount of autogenous bone. However, fibula grafting could be avoided by the grafting of both Neobone and autogenous bone. Although further careful observation of the course is necessary, favorable bone formation was observed. This grafting of both Neobone and autogenous bone may also be useful for preventing stress shielding in joint replacement with tumor prostheses.

## Consent

Written informed consent was obtained from a relative of the patient for publication of this case report and any accompanying images. A copy of the written consent is available for review by the Editor-in-Chief of this journal.

## Competing interests

The authors declare that they have no competing interests.

## Authors' contributions

YY wrote the manuscript, SO contributed to the manuscript, YT critically reviewed the manuscript. All authors read and approved the manuscript.
